# Patients’ experience of care index: A new, reliable, and useful questionnaire in lumbar spine surgery

**DOI:** 10.1016/j.bas.2024.104140

**Published:** 2024-11-17

**Authors:** Arkan Sam Sayed Noor, Björn Knutsson

**Affiliations:** a Clinical Sciences Department, College of Medicine, University of Sharjah, University City Rd, University City, Sharjah, United Arab Emirates; b Department of Diagnostics and Intervention (Orthopaedics), Umeå University, 901 87, Umeå, Sweden

**Keywords:** Spine surgery, Perception of care, Patient satisfaction, Index, Reliability

## Abstract

**Introduction:**

Patient's experience of care (PEC) is crucial in enhancing and sustaining healthcare quality.

**Research question:**

the primary aim of this study is to establish and assess a new questionnaire index designed to measure PEC following elective spinal surgery. This index serves as a tool to document, enhance, and maintain the quality of healthcare provided in this context.

**Material and methods:**

The studied PEC index comprises 7 questions, each addressing different aspects of perioperative care. Our study involved 300 post-spinal surgery patients, sourced from the Swedish national register for spine surgery. Collected data included age, gender, education level, self-rated health, and primary language. The patients were contacted via telephone by trained interviewers, approximately 35 days after their surgery (with 60 patients re-interviewed after a few weeks). We employed linear regression, *t*-test, and ANOVA models to examine the associations between the PEC index and the documented variables.

**Results:**

The PEC index demonstrated good internal consistency and reliability (Cronbach alpha = 0.76, interclass correlation coefficient = 0.87). Additionally, the utility measures indicated associations between the PEC index and factors such as higher age (p = 0.014), male gender (p = 0.012), and better self-rated health (p = 0.011).

**Discussion and conclusion:**

The PEC index seems to be a promising tool with a clinically useful composite questionnaire for assessing PEC in patients undergoing elective spine surgery. In clinical settings, the index can accompany other outcome scores to evaluate and compare different diagnoses and management methods.

## Highlights

• Patient's experience of care (PEC) is crucial in enhancing healthcare quality.
• The studied PEC index seems to be a promising tool with a useful questionnaire.
• Better postoperative outcome was associated with higher age and male gender.
• Worse postoperative outcome was associated with fair and poor self-rated health.

## 1 Introduction

Patient's experience of care (PEC) plays a crucial role in enhancing and sustaining healthcare quality (Beattie et al., 2015).^1^ Several surveys have underscored the significance of measuring PEC, primarily utilizing qualitative methods (Beattie et al., 2015; Knutsson et al., 2022a, Knutsson et al., 2022b; Wilde et al., 1993).^1-3)^ Qualitative studies are valuable for capturing aspects of PEC that quantitative data might miss and, therefore can serve as a foundation for developing quantitative instruments for experience, satisfaction, and outcome measures (Beattie et al., 2015; Wilde et al., 1993, 1994).^1,3,4)^ However, findings from qualitative studies cannot always be generalized, making it challenging to compare results across different centers and countries, and to analyze changes in PEC over time or after implementing quality improvement. To measure PEC, numerous national instruments have been developed worldwide (Beattie et al., 2015).^1^ Despite their common use in clinical practice, concerns have arisen regarding certain questionnaires' usefulness, validity, reliability, cost-effectiveness, acceptability, and educational impact (Wilde Larsson and Larsson, 2002; Van der Vleuten, 1996).^5,6)^ Furthermore, patients' healthcare ratings may be influenced by age, gender, education level, self-rated health, ethnicity, and primary language. Consequently, adjusting for the case mix is vital when comparing hospitals and countries (Elliott et al., 2001).^7^).

In the context of spine surgery, PEC has predominantly been accessed using global rather than multidimensional instruments, with most studies focusing on surgical outcomes rather than PEC (Menendez et al., 2019).^8)^ The Swedish National Register for Spine Surgery (Swespine) is a prospective registry financed by the Swedish Association of Local Authorities and Regions (SALAR) (Stromqvist et al., 2013).^9)^ The Swespine's annual reports include baseline characteristics, surgical data, outcome measures, and two questions about PEC. The 2014 annual report analyzed PEC related to spine surgery through collaboration between Swespine and the survey company, the Indikator Institute (Stromqvist et al., 2014).^10)^ The initial questionnaire included seven questions about PEC. The categorical answer options were registered on a Likert scale. However, the limitation of using categorical scales suggested the need for a continuous variable for the total PEC that would allow for better comprehension and a more sensitive analysis over time and between hospitals and countries.

The main objective of this study was to develop a reliable and comprehensive index encompassing various crucial dimensions of PEC in elective lumbar spine surgery. Additionally, we sought to explore any associations between the PEC index and factors such as age, gender, education level, self-rated health, or primary language.

## 2 Material and Methods

### 2.1 Population

Between the September 1, 2015 and the October 21, 2015, 300 consecutive patients from Sweden were enrolled in this study. Throughout this period, the Indikator Institute was notified whenever a patient underwent an elective lumbar spine procedure and was registered in Swespine. Trained interviewers phoned the patients and obtained their informed consent to participate in the study, and all patients agreed to participate. The inclusion criteria for the study required registered patients to have a diagnosed lumbar spine condition treated surgically, be between 18 and 90 years of age, and be fluent in Swedish.

The median time between discharge and interview was 35 days (range, 24–61). To assess the test-retest reliability of the questionnaire, 60 out of the 300 patients were re-interviewed 2–3 weeks after the initial interview. The questionnaire used in the interviews comprised seven specific questions about PEC.

**Fig. 1:**
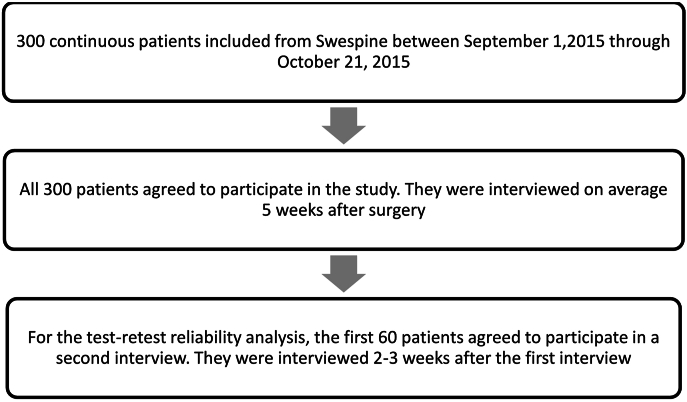
Flow diagram for the study population.

Fig. 1 shows a flow diagram for the study.

### 2.2 Patients’ experience of care index

The questionnaire's development involved a collaboration between the Indikator Institute and a group of spine surgeons (Knutsson et al., 2022a, Knutsson et al., 2022b).^11)^ The Indikator Institute has been engaged in patient-experienced healthcare follow-up since the early 2000s and has established validated survey tools for postal patient questionnaires in collaboration with international research experts, patient groups, and healthcare professionals. The Indikator Institute has vast experience in capturing patient experiences through various methods, including postal questionnaires, telephone interviews, focus groups, interactive survey stations, and observational studies (Stromqvist et al., 2014; Indikator, 2024).^10,12^).

The PEC index comprises seven questions representing important care domains (physicians and other healthcare professionals' conduct, pain control, involvement, discharge information, and overall evaluation of the care) (Menendez et al., 2019; Lehrich et al., 2021). Each question is assigned a value within the range of 0–1, and the sum of the seven questions is divided by 7 to calculate the PEC index. To simplify reporting, the results are presented as whole numbers within the 0–100 range, where higher values indicate a more positive experience.

**Fig. 2:**
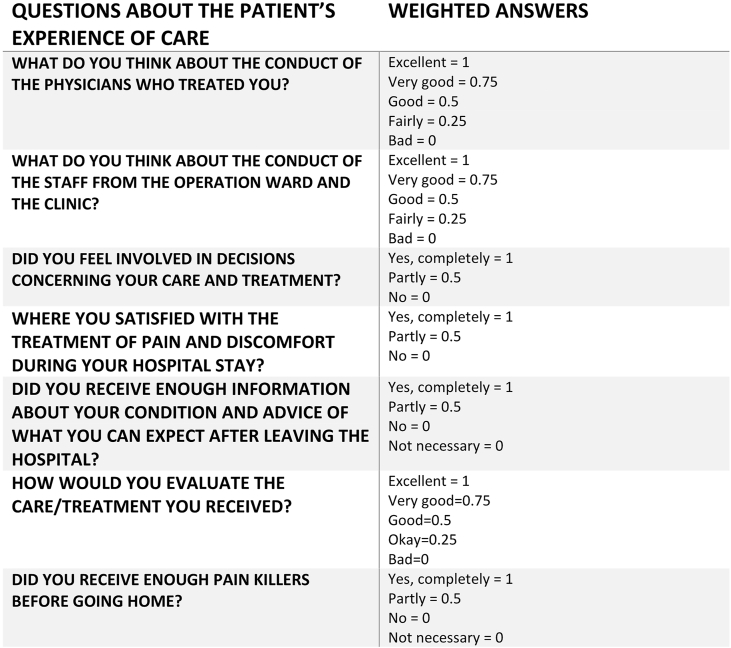
Overview of the PEC Index questions and their weighted answers.

Fig. 2 provides an overview of the questions and their weighted answers.

### 2.3 Swespine

The Swespine is a Swedish prospective registry financed through the SALAR (Stromqvist et al., 2013). Over 10.000 spinal surgical procedures are registered each year, with an approximate completion rate of 85%. Preoperatively, demographic data and functional status parameters such as age, sex, smoking habits, sick leave, working status, earlier spinal surgery, the Oswestry Disability Index (ODI), the EQ-5D quality of life index, and back and leg pain are measured on the numeric rating scale (NRS). The surgeon recorded surgical data, including diagnosis, surgical procedure, and complications. Follow-up questionnaires at 1 year are sent to the patients along with a prepaid, addressed return envelope.

### 2.4 Statistical analysis

Statistical calculations were conducted using SPSS version 27.

The PEC index is presented as the mean with 95% confidence intervals (95% CI).

A Cronbach alpha analysis was used to measure the PEC index's internal consistency, which shows how the included items are related as a group. To assess test-retest reliability between the first and second interviews, an interclass correlation coefficient (ICC) analysis was employed. The ICCs were calculated using absolute agreement on a two-way mixed model and reported with 95% CI. A paired *t*-test was also utilized to describe the agreement between the first and second interviews. The mean from the paired t-tests is reported along with a 95% CI.

For the utility analysis and to provide a recommended minimal sample size for future studies, a two-sided power analysis was performed. The standard error of the mean (SEM) was used to calculate the minimal detectable change (MDC). The MDC is equal to 1.96 x SEM x square root of 2. The standard deviation (SD) from the test-retest reliability cohort was used (SD = 16), along with a significant level of 0.05, and a power of 0.8.

To explore potential associations between the PEC index and age, gender, education level, self-rated health, and primary language, unadjusted bivariate analyses were conducted. Linear regression, *t*-test, or ANOVA models were applied depending on the variable analyzed.

## 3 Results

The SEM was 1.06 and the MDC was 3.

The power analysis, according to Bonett (2002),^13)^ revealed that a group size of 896 participants would be required to detect a difference of 3, 162 participants would be required to detect a difference of 5, and 42 participants would be sufficient to detect a difference of 10.

A total of 300 patients took part in the first interview, and among them, the first 60 patients underwent a second interview 2–3 weeks later for the test-retest reliability evaluation. Of the 300 patients, 296 (99%) completed all the questions, yielding a mean PEC index of 88 (95% CI, 86–89).

The characteristics of the 300 patients are described in Table 1.

**Table 1:** Characteristics of inclusion for the participants.

Characteristic	N = 300
**Age in years, mean (SD)**	63 (15)

**Sex, n (%)**	
**Female**	157 (52)
**Male**	143 (48)

**Diagnosis, n (%)**	
**Lumbar spinal stenosis**	208 (69)
**Lumbar disc hernia**	45 (15)
**Chronic low back pain**	22 (7)
**Isthmic spondylolisthesis**	15 (5)
**Other diagnosis**	10 (3)

**Level of education, n (%)**	
**University graduate**	84 (28)
**High school graduate**	135 (45)
**Compulsory school (9 years)**	81 (27)

**Self-rated health, n (%)**	
**Excellent**	51 (17)
**Very good**	79 (26)
**Good**	104 (35)
**Fair**	50 (17)
**Poor**	16 (5)

**Primary language, n (%)**	
**Swedish**	284 (95)
**Other than Swedish**	16 (5)

The internal consistency of the seven questions was tested, resulting in a Cronbach alpha of 0.76. Moreover, the test-retest analysis demonstrated good to excellent reliability, with an ICC of 0.87 (95% CI, 0.80–0.92) between the PEC index calculated from the first and second interviews. The mean PEC index was 87 (SD 16) in the first interview and 86 (SD 17) in the second interview. The paired *t*-test analysis showed a difference of 1 (95% CI, −1 to 3) between the first and second interviews.

The association between the PEC index and the studied variables (age, gender, self-related health, university degree, and Swedish as the primary language) is shown in Table 2.

**Table 2:** Results from bivariate analyses between age, sex, education level, self-rated health, primary language, and PEC index.

Characteristic	PEC index	p-value
**Age (continuous)**	B = 0.137 (0.028–0.246)^a^	0.014∗

	Mean (95 % CI)	

**Sex**		0.012∗∗
**Female**	86 (83–88)	
**Male**	90 (88–92)	

**Level of education**		0.011∗∗∗
**University graduate**	90 (89–93)	Reference (ref)
**High school graduate**	85 (83–88)	0.004; ref
**Compulsory school (9 years)**	89 (86–91)	0.346; 0.067

**Self-rated health**		<0.001∗∗∗
**Excellent**	92 (87–96)	ref.
**Very good**	93 (90–95)	0.601; ref
**Good**	87 (85–90)	0.058; 0.005; ref
**Fair**	80 (76–85)	<0.001; <0.001; 0.003; ref
**Poor**	78 (71–86)	<0.001; <0.001; 0.009; 0.054

**Primary language**		0.713∗∗
**Swedish**	88 (86–90)	
**Other than Swedish**	87 (80–94)	

## 4 Discussion

Although there is some conceptual overlap between PEC and degree of satisfaction with care, these two terms are often used interchangeably in the literature. Additionally, satisfaction is usually measured as quantitative multidimensional points on Likert scales (Menendez et al., 2019; Gutiérrez-Sánchez et al., 2020).^8,14,15)^ However, the concept of PEC in clinical settings requires further elaboration and expansion, including validation and reliability testing. For instance, Menendez et al. (2019) conducted a systematic review which found that most evaluation surveys measuring patient satisfaction focused on outcomes (96.2%) rather than the experience of care (1.9%). The authors stressed the importance of developing an instrument to measure patient satisfaction in the field of spine surgery because this type of instrument's widespread use could aid in assessing the effectiveness of surgical intervention for spinal pathology.

In the present study, the primary finding was that the PEC index proved to be a reliable and useful measure for capturing PEC in elective spine surgery. The questionnaire's development involved collaboration with the Indikator Institute, which has extensive experience in conducting similar surveys in both Swedish and international contexts, including studies on patients who had undergone degenerative lumbar spine surgery (Knutsson et al., 2022a, Knutsson et al., 2022b).^11)^ The contents of the PEC index (physicians and other healthcare professionals' conduct, pain control, involvement, discharge information, and overall evaluation of the care) are often evaluated and correlated to patients' satisfaction. Among others, Levin et al. (2018) ^16)^, Bible et al. (2018) ^17)^, and Ronnberg et al. (2007) ^18)^ have shown a relationship between the counseling or bedside manner of the treating physician/staff and patient-reported satisfaction. Respect, friendliness, time, attentiveness, and involvement are a few characteristics patients expect every healthcare provider to manifest. On the other hand, some studies linked satisfaction to peri-operative pain scores, either as absolute values or as changes from pre-to postoperative pain. For instance, Menendez et al. (2019)^15)^ reviewed the literature to report significant associations between lower Visual Analogue Scores, Numerical Rating Scales, and other pain scales and higher satisfaction rates, and vice versa.

We observed associations between the PEC index and age, gender, and health status, concurring with the results of a systematic review published by Lehrich et al. (2021)^19)^ The authors studied the spine surgery literature to analyze factors predictive of patient satisfaction as measured by the Press Ganey and Hospital Consumer Assessment of Healthcare Providers and Systems surveys, both commonly used in the United States of America. Among other results, older age and male gender correlated to better satisfaction, while worse overall health was a factor associated with worse patient satisfaction (Shabat et al., 2005; Mets et al., 2020; Gepstein et al., 2006).

The quantitative nature of the PEC index enables its easy administration in large national and international surveys and registers such as the Swedish Population Survey. This measure helps detect patient's experiences with quality improvements and allows for comparisons within and among different healthcare providers. The scoring system of the PEC index is explicitly defined and straightforward to calculate. The low number of missing answers and high response rate indicate high acceptability and responsiveness with minimal nonresponse bias (Fincham, 2008).

The index can be considered a composite score that can act as a surrogate for PEC parameters. Therefore, the index can accompany other outcome scores, such as functional outcome and quality of life measures. In a clinical setting, the index can be used to evaluate and compare different diagnoses and management methods. In Sweden, for instance, PEC is now required as a routine measure included in Swespine registry to evaluate postoperative outcomes and justify financial allocation.

The current study's strength lies in its data collection method and the high response rate achieved for the questionnaire. All eligible patients willingly participated in the survey, with 99% (296/300) completing the questionnaire. Furthermore, in the test-retest reliability analysis, all participants (60/60) completed the questionnaires in the first and second interviews.

Notably, our study has some limitations. First, the validity of these kinds of surveys can be challenging to assess. One way, as emphasized by Juniper et al. (2005, 2010), was to conduct parallel form validity analysis, i.e. to divide our set of questions into two equivalent sets (forms), where both sets contain questions that measure the same construct, knowledge, or skill and then to estimate its validity. Unfortunately, this was not done in the current study. Another limitation is the questionnaire's brevity, with only seven questions and no way for participants to rank the questions' importance from their perspective. Although additional questions may enhance the internal consistency of a questionnaire, they could also lead to a lower response rate, a higher percentage of missing answers, and a longer time to complete the assessment. Nevertheless, the high participation and response rate indicate the importance of our questions for the patients. Another limitation is the wide range of ages (18–90 years) included in the study, with no scores indicating the patient's mental status. However, patients with mental impairment rarely become candidates for elective spinal surgery. Therefore, we anticipate that the eventual number of mentally impaired patients is negligible.

## 5 Conclusion

The PEC index seems to be a promising tool with a clinically useful composite questionnaire for assessing PEC in patients undergoing elective spine surgery. In clinical settings, the index can accompany other outcome scores, such as functional outcome and quality of life measures to evaluate and compare different diagnoses and management methods.

## Informed consent statement

Patient consent was obtained from all patients.

## Author contributions

Both authors contributed equally to the conceptualization, methodology, validation, formal analysis, data curation, and writing of original draft preparation.

## Institutional review board statement

The study was conducted according to the guidelines of the Declaration of Helsinki, and approved by the local Ethics Committee of Umeå University.

## Data availability statement

The database will be available upon reasonable request to the corresponding author. The statistical analysis plan and syntax will be made available upon request.

## Declaration of competing interest

The authors declare that they have no known competing financial interests or personal relationships that could have appeared to influence the work reported in this paper.
